# Targeting *KRAS G12C* mutations in colorectal cancer

**DOI:** 10.1093/gastro/goac083

**Published:** 2022-12-30

**Authors:** Ming-He Zhao, Ai-Wen Wu

**Affiliations:** Key Laboratory of Carcinogenesis and Translational Research, Ministry of Education; Unit III, Gastrointestinal Cancer Center, Peking University Cancer Hospital & Institute, Beijing, P. R. China; Key Laboratory of Carcinogenesis and Translational Research, Ministry of Education; Unit III, Gastrointestinal Cancer Center, Peking University Cancer Hospital & Institute, Beijing, P. R. China

**Keywords:** KRAS, G12C, drug resistance, colorectal neoplasms, combination therapy

## Abstract

With the advent of Kirsten rat sarcoma viral oncogene homologue G12C (KRAS G12C) inhibitors, RAS is no longer considered undruggable. For the suppression of RAS, new therapeutic approaches have been suggested. However, current clinical studies have indicated therapeutic resistance after short-lived tumour suppression. According to preclinical studies, this might be associated with acquired genetic alterations, reactivation of downstream pathways, and stimulation for upstream signalling. In this review, we aimed to summarize current approaches for combination therapy to alleviate resistance to KRAS G12C inhibitors in colorectal cancer with a focus on the mechanisms of therapeutic resistance. We also analysed the relationship between various mechanisms and therapeutic resistance.

## Introduction

Mutations in *RAS*, one of the most common human oncogenes, are found in 27% of human cancers [1]. Three homologues of the human *RAS* gene family have been identified: neuroblastoma *RAS* viral oncogene homologue (*NRAS*), Harvey rat sarcoma viral oncogene homologue (*HRAS*), and Kirsten rat sarcoma viral oncogene homologue (*KRAS*). These homologues encode four distinct tumour-causing guanosine triphosphatases (GTPases): *NRAS*, *HRAS*, *KRASa*, and *KRASb*; of these, *KRASa* and *KRASb* result from alternative splicing. As a switch protein, RAS cycles between an active guanosine triphosphate (GTP)-bound and inactive guanosine diphosphate (GDP)-bound state. A small GTPase from the RAS super protein family is encoded. KRAS has a molecular weight of 21.6 kDa and contains 188 acids. It is a guanine nucleotide-binding protein with GTPase activity, which can combine with GTP, GDP, and GTP enzyme. It mainly comprises three regions: G-domain, C-terminal, and C-terminal CAAX boxes. The G-domain, which contains Switch I and Switch II rings, is a highly conserved domain responsible for GDP–GTP switching [2]. The C-terminal, including the CAAX box, differs significantly between RAS family members. Notably, KRAS protein has a high affinity with GDP and GTP [3], acting as a molecular switch that cycles between the inactive state of GDP-binding protein and active state of GTP-binding protein [4]. In the active state, RAS transmits signals from the cell membrane to the nucleus, playing a crucial role in cellular proliferation, differentiation, and survival by meditating numerous downstream pathways, including the rapidly accelerated fibrosarcoma (RAF)/mitogen-activated protein kinase (MEK)/extracellular-regulated kinase (ERK) and phosphoinositide-3-kinase (PI3K)/AKT/mTOR pathways [5]. The PI3K/AKT/mTOR signal can promote the growth of tumour and increase the risk of metastasis by meditating epithelial–mesenchymal transition (EMT) [6, 7]. Additionally, the activated pathway can affect other oncogenes, such as doublecortin-like kinase (DCLK1), G protein-coupled receptor 56 (GPR56) and tripartite motif-containing 59 (TRIM59), and promote the migration and invasion of colorectal cancer (CRC) cells [8, 9]. RAS and its downstream effectors are believed to be attractive targets for cancer therapy because of their critical role in driving the hallmarks of cancer and the frequency of mutations detected in human cancer.

**Figure 1.**
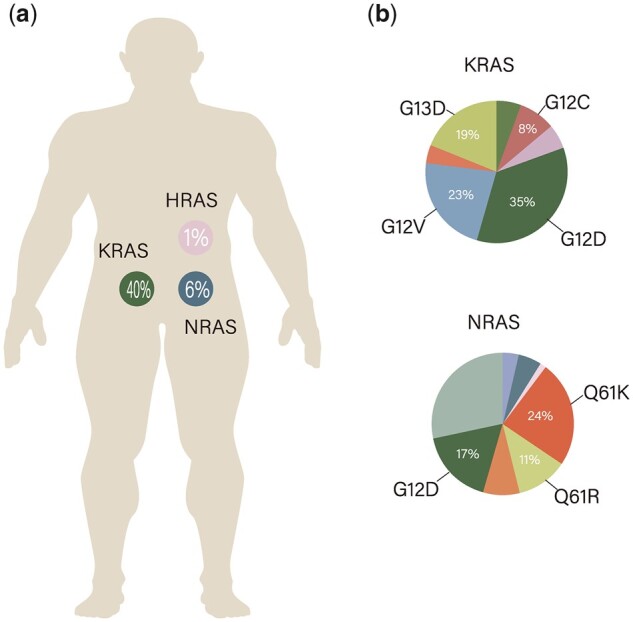
Frequency of different *RAS* mutations in colorectal cancer. (a) Distribution of *RAS* isoform mutations in colorectal cancer. (b) Frequency of mutated amino-acid substitutions at the mutated isoform in colorectal cancer. Data obtained from the Cancer Genome Atlas (PanCancer) from cBioportal and Catalogue of Somatic Mutations in Cancer v95. *NRAS*, neuroblastoma *RAS* viral oncogene homologue; *HRAS*, Harvey rat sarcoma viral oncogene homologue; *KRAS*, Kirsten rat sarcoma viral oncogene homologue.

Among these genes, the most oncogenic *RAS* mutations in humans are found in *KRAS*, which represents 85% of all mutations [1]. According to the data from TCGA PanCancer Atlas Studies, non-small-cell lung cancer (NSCLC), CRC, and pancreatic cancer [particularly, pancreatic duct adenocarcinoma (PDAC)] are the three most common cancers associated with *KRAS* mutations, with incidence of ∼30%, ∼42%, and ∼80%, respectively [10–12]. Oncogenic mutations in *KRAS* mostly focus on Codons 12, 13, and 61. The levels of mutation at these sites vary depending on the isoform and malignancy type. In particular, glycine residues at Codon 12 are most frequently affected by missense mutations [1]. Moreover, origin tissues can predict *KRAS* missense mutations; *KRAS G12D* accounted for ∼25%–40% of all *KRAS* mutations in CRC and PDAC, whereas *KRAS G12C* accounted for >40% of all *KRAS* mutations in lung adenocarcinoma [10, 12, 13] (Figure 1). The patients with mutant-*KRAS* CRC, with a median overall survival of ∼2 years, had worse overall survival progression and poorer prognosis than those with wild-type-*RAS* CRC [14, 15]. As the first intracellular messenger of epidermal growth factor receptor (EGFR), KRAS circulates between the inactive GDP-binding protein and active GTP-binding protein [13, 16]. Meanwhile, missense mutations at Codons 12, 13, and 61 block GTP hydrolysis (i.e. block progression to the inactive stage) by limiting the interaction of GTPase-activating protein (GAP) proteins with the GTPase site of RAS [17]. Subsequently, KRAS triggers multiple proliferative signalling pathways, including the mitogen-activated protein kinase (MAPK)/ERK and PI3K pathways, to promote cell growth, division, and differentiation [18–20]. Thus, there is a critical need to establish an effective target therapy to sustainably inhibit the reactivity of KRAS and its downstream signalling among patients with this mutation. Unfortunately, despite 40 years of research on this topic, no treatment has proven stable enough to permanently suppress *KRAS* mutations. Further, no drugs have shown efficacy while remaining within acceptable cytotoxic levels. Because of its affinity with GTP and the absence of significant binding sites that could accommodate allosteric inhibitors, *KRAS* has long been considered ‘undruggable’ [21, 22]. As mentioned previously, for patients with *KRAS* mutation, treatment with EGFR or vascular endothelial growth factor (VEGF) inhibitors even combined with chemotherapy has not shown any benefit.

KRAS G12C inhibitors have demonstrated completely conceptual improvement in patients with *KRAS* mutation [23]. In addition, this special targeted inhibitor has a significant influence on mutated solid cancer cells. Herein, KRAS G12C inhibitors have drastically altered the behaviour of mutant cells in CRC. Further, combined therapy with other inhibitors has shown a potential inhibitory effect in CRC. Optimizing the use of combination therapy to reduce acquired resistance is vital.

## Limited efficiency of KRAS G12C inhibitors

Recent attempts to suppress KRAS inhibitors that are specific to mutant subtypes have proven successful. The foundational work for therapeutic KRAS blockade was laid by a ground-breaking discovery of binding of a KRAS G12C inhibitor to the Switch II region [24]. The potential target is significant across all patients with mutant *KRAS* who have limited effective treatment therapies. The first KRAS G12C inhibitor targeting the inactive GDP-bound state was ARS-853, which led to the development of novel anti-RAS therapeutics [25]. Then the same team led by Janes *et al.* [26] developed the first G12C inhibitor *in vivo* known as APS1620, which overcame the shortcomings of ARS-853 regarding plasma stability and oral availability, showing higher potency and selectivity for KRAS G12C. Moreover, among several compounds with improved biological activities, adagrasib (MRTX849) and sotorasib (AMG-510) were the first drugs to enter clinical trials [23, 26, 27]. The trials revealed that these two drugs can reduce the viability of mutant-*KRAS* cells and inhibit MAPK-pathway signalling by interacting with the Switch II region [23, 27, 28]. Notably, among patients with NSCLC, 19 (32.2%) showed a confirmed objective response, whereas 52 (88.1%) achieved disease control [28]. The effectiveness and tolerability of sotorasib in patients with advanced or metastatic NSCLC (NCT03600883) were evaluated in the recently completed phase II CodeBreak 100 trial [29]. This trial included patients who failed at least two previous anticancer therapies and indicated that the drug was safe and tolerable, similarly to the previously reported phase I data [28]. NSCLC cases had a median progression-free survival (mPFS) of 6.3 months, disease-control rate (DCR) of 88.1%, and overall response rate (ORR) of 36%. Meanwhile, the clinical trial (NCT03785249) of adagrasib is currently ongoing. The preliminary findings have demonstrated the effectiveness and tolerated drug toxicity of adagrasib, with ORR and DCR of 45% and 96%, respectively, in NSCLC.

G12C variants only account for 3% of CRC cases, indicating that they are not as abundantly expressed as observed in cases of pancreatic cancer or NSCLC [30]. For these cases, the efficacy of the KRAS G12C inhibitor was comparable to that reported in a previous study. Among patients with a subtype of CRC, 7.1% (three patients) had a confirmed response, with an mPFS of 4 months after treatment with sotorasib [28]. The data presented at the 2020 EORTC-NCI-AACR Annual Symposium supported the efficacy of adagrasib in CRC, with an ORR of 17% and DCR of 96% [31]. However, some patients experienced disease progression immediately after an initial response [28]. In addition, the clinical activity of the drug in CRC cases was reduced compared with that in NSCLC cases. These results suggest that targeting KRAS G12C should not be the only therapeutic strategy employed in a study. Combination therapy can be used for limiting adaptive feedback, similar to the experience with v-raf murine sarcoma viral oncogene homolog B1 (BRAF) inhibitors; this is essential to maximize the durability of response in each tumour type [32, 33]. Importantly, *KRAS G12C* mutation plays a key role in colon carcinogenesis and development compared with other tumours; however, the effect of specific inhibitors remains unclear. Further research on the molecular pathways associated with *KRAS G12C* mutation is warranted and it is important to determine the mechanism by which various tumours respond to inhibitors. Moreover, the mechanisms of resistance to KRAS inhibitors are complex and the choice of the combination therapy needs further investigation to guide future treatment approaches.

## KRAS G12C resistance mechanisms and co-targeting combination

### Mechanisms of *KRAS* gene alteration

Preclinical studies have indicated that the therapeutic efficacy of KRAS G12C inhibitors is reduced via various potential resistance mechanisms, including innate, acquired, and adaptive tumour responses. A lack of dependency on KRAS signalling could partially account for the intrinsic resistance noted in preclinical models, which may underlie the variations in patient responsiveness [23, 34, 35]. In some non-KRAS-dependent cells, even when KRAS was completely inhibited, the cells were still viable, indicating that the resistance of some *KRAS G12C-*mutated tumours to G12C inhibitors is due to the low dependence of cells on KRAS [36].

Variation in mutant-*KRAS* cells may impact the therapeutic potential of KRAS G12C inhibitors. Because these inhibitors only inhibit KRAS in the inactive GDP-bound state, Xue *et al*. [16] attributed treatment resistance to the variation in *KRAS*-mutant cells. However, cells in the GTP-bound state are not sensitive to inhibitor treatment and continue to activate downstream signalling pathways, which in turn promote cell proliferation and differentiation. This may be attributed to the heterogeneous response of *KRAS G12C*-mutant cells to G12C inhibition by ARS-1620. The newly formed KRAS G12C remains in its active GTP-bound, drug-insensitive state under the action of EGFR and phosphatase SHP2 signalling. Upon further investigation regarding the factors responsible for the evasion of KRAS G12C suppression, heparin-binding EGF (HBEGF) and aurora kinase (AURKA) were found to play a significant role in KRAS treatment resistance through the analysis of differential expression and genome-wide knock-down screens. HBEGF potentially enhances mutant cell resistance to KRAS G12C inhibitor mediated through the EGFR pathway. HBEGF was transiently downregulated in response to ARS-162 but was rapidly upregulated after 48 h. Furthermore, silencing of HBEGF using small interfering RNA significantly enhanced the inhibitory effect of the KRAS G12C inhibitor. Moreover, AURKA may be required for reversing drug-induced quiescence; this maintains KRAS in the active GTP-bound state, which when stabilized reacts with KRAS G12C and the downstream effector CRAF. Meanwhile, there is a difference in the operating sequence of HBEGF and AURKA; HBEGF exposure is the initial stimulus and AURKA operates later. Although these experiments were verified in lung cancer cells, they also have implications for CRC promoting. Thus, treatment resistance can result from the non-uniform stage of *KRAS G12C*-mutant cells.

The activation of wild *RAS* could also explain the resistance to specific inhibition strategies. Ryan *et al*. [37] studied the adaptive response of *KRAS G12C*-mutant lung, colon, and pancreatic cancer cells to selective KRAS G12C inhibition using the covalent KRAS G12C inhibitors ARS-1620 and AMG 510. They revealed that KRAS G12C inhibitors downregulated the MAPK pathway in all cells, as evidenced by the decreased expression of phosphorylated MEK, ERK, and receptor tyrosine kinases (RTKs) at 4 h. Despite continuous suppression of GTP-bound KRAS, MAPK-pathway reactivation was observed in most cells by 24–48 h. Isoform-specific pull-down assays revealed that treatment with KRAS G12C inhibitor resulted in a several-fold increase in NRAS GTP and HRAS GTP levels. These results suggest that *KRAS G12C*-mutant cells rapidly adapt to the selective suppression of mutant *KRAS* caused by activating wild-type *RAS*; further, these indicate that this oncogenic bypass was sufficient for restoring MAPK signalling. Meanwhile, Zhao *et al*. [38] discovered the genetic basis of the action of first-line mutant GTPase inhibitor sotorasib in samples with *KRAS* mutation. The resistance to KRAS (G12C) inhibition is associated with low-allele-frequency hotspot mutations in preclinical patient-derived xenograft and cell models. In the single-cell sequencing of isogenic lineage, including *KRAS* (*G12V* or *G13D*), *NRAS* (*Q61K* or *G13R*), and *BRAF* (*G596R*), they could identify either or both secondary *RAS* and *BRAF* mutations (*G12C*) in the same cells. Further, the novel mutations could bypass the inhibition without affecting the original inactivation subgroup. This study demonstrated a heterogenous pattern of resistance with multiple sub-clonal events during G12C-inhibitor treatment.

In addition to the generation of novel KRAS and activation of wild-type informs, multiple co-mutational alterations can cause treatment resistance. Awad *et al*. [39] analysed *KRAS G12C*-mutant cells among patients who received adagrasib monotherapy. They reported putative mechanisms causing treatment resistance at the genomic and histologic levels. Among 38 patients with *KRAS G12C*-mutated tumours, 17 developed mutational resistance mechanisms; of 7 (18% of the cohort) patients with multiple co-mutational mechanisms, 4 (11%) had CRC. Patients with CRC had multiple genetic alterations, including acquired *KRAS* mutations in *G12D/R/V*, *G13D*, and *H95R*, as well as amplified KRAS G12C alleles. Other notable findings were the presence of multiple carcinogenic fusions involving *BRAF*, *NRF1*, and *FGFR3*. The acquired bypass gene mechanism included *MET* amplification and loss of function mutations in *NF1* and *PTEN*.

Regarding the adaptive mutations of *KRAS*, different mutant sites have various degrees of resistance to KRAS inhibition. Koga *et al*. [40] identified 12 different secondary *KRAS* mutations and reported that 124 (87%) individuals had secondary *KRAS* mutations. For instance, *Y96D* and *Y96S* were resistant to the individual inhibitors, i.e. a new SOS1 inhibitor (BI-3406) and trametinib; however; the combination of the two inhibitors was highly effective against both mutations. *G13D*, *R68M*, *A59S*, and *A59T*, which were extremely resistant to sotorasib, were found to be sensitive to adagrasib. *Q99L*, which was resistant to adagrasib, remained susceptible to sotorasib. Tanaka *et al*. [41] investigated acquired resistance to adagrasib using circulating free DNA (cfDNA). The identical KRAS G12C and TP53 F338fs variants that appeared in patients’ tumours and cfDNA before therapy were also detected after the emergence of resistance along with the introduction of 10 unique mutations affecting the four *RAS/MAPK* subunits, namely *KRAS*, *NRAS*, *BRAF*, and *MAPK1*. In addition, the three *KRAS* mutations *G13D*, *G12V*, and *Y96D* were detected in the cfDNA after advancement. Structural modelling revealed that the *Y96D* mutation destroys crucial hydrogen bonds between *KRAS* and the *Y96D* residues of adagrasib. *Y96D* was resistant not only to adagrasib but also to sotorasib and ARS-1620. The presence of a Switch II pocket-type mutation in *Y96D* may hinder the binding of KRAS G12C (OFF) inhibitors and promote tolerance to KRAS G12C (ON) inhibitors. However, this can be inhibited by a functionally unique KRAS G12C (ON) inhibitor (RM-018). Although the experiment mainly involved patients with lung cancer, differences among secondary mutant sites may similarly contribute to treatment resistance in CRC.

## Bypass signalling resistance mechanism

### MAPK-pathway reactivation

The bypass MAPK pathway plays an important role in KRAS therapy as a downstream component of the RAS cascade. Similar to BRAF and EGFR, KRAS G12C inhibitors are the upstream targets of the MAPK pathway. Regarding possible mechanisms of therapeutic resistance, feedback activation of the MAPK pathway has an impact on the efficacy of specific inhibitors [16, 23, 27, 42]. Hence, this feedback activation may cause resistance of KRAS to specific allele inhibition.

As previously observed in *BRAF*-mutated CRC [43], the adaptive response to RAF-inhibitor treatment was a useful reference for the development of KRAS G12C inhibitors. *BRAF V600E*-mutated cells treated with RAF inhibitors can result in a rapid relief of upstream feedback of RTKs and RAS. Furthermore, the dimerization of BRAF can decrease the sensitivity to RAF inhibitors and lead to the reactivation of ERK signalling [44]. Hence, combination therapies that target multiple nodes in the pathways (especially upstream nodes) can induce a more marked response, as observed in preclinical models. To confirm this hypothesis, Amodio *et al*. [45] examined the effects of AMG510 in CRC cells. The levels of cell basal RTK activation in CRC cells were higher and more responsive to growth factor stimulation than those in NSCLC cells. Moreover, the rapid rebound of RTKs, especially EGFR, was responsible for CRC resistance to KRAS G12C inhibitors. Combination therapy targeting both EGFR and KRAS G12C was highly effective in CRC cells. The combination of cetuximab and sotorasib effectively inhibited the activation of the EGFR-driven MAPK pathway in CRC cells, thus sustaining downstream target inhibition, significantly improving KRAS G12C inhibition, and achieving tumour regression.

The aforementioned studies demonstrate that the reactivation of RTK signalling (especially EGFR) is an important cause of treatment resistance to KRAS G12C inhibitors in CRC. Meanwhile, in patients with *EGFR* wild-type CRC, *KRAS* mutations often lead to secondary resistance resulting in inferior outcome of anti-EGFR therapy [46]. Thus, targeted therapies against EGFR have been investigated as they provide a more effective direction in clinical practice. Cetuximab monotherapy has shown favourable clinical results in *EGFR*-mutated lung cancer [47, 48]. Further, a combination of cetuximab and fluorouracil chemotherapy has shown good results in CRC [49]. In *BRAF*-mutated CRC, cetuximab along with BRAF inhibitors is already the standard second-line treatment in clinical practice [43, 50]. Currently, the combination of KRAS G12C and EGFR inhibitors has shown positive results in mouse models [16, 27].

*In vivo* and *in vitro* experiments have demonstrated the reactivation of upstream RTKs, which may be critical for the resistance to KRAS inhibition in CRC. Furthermore, compared with NSCLC, the pronounced dependency on the EGFR/MAPK pathway in CRC may be responsible for differences in their treatments. The possible reasons why EGFR triggers resistance to KRAS G12C inhibitors are multiple. It may be related to the intrinsic RTK dependency and sensitivity of CRC, high active RTKs levels, and reactivation of downstream effectors. In these cases, a combination of KRAS inhibition and EGFR blockade can be a viable therapeutic strategy. Nevertheless, rational and tissue-specific combination therapies are necessary for precise and effective disease control. The characteristics of other *KRAS-*mutant alleles varied extensively; thus, further investigation is warranted to determine whether other alleles can benefit from EGFR-blockade combination therapy.

SHP2 plays an important role in the RAS pathway mediated by RTK phosphorylation, providing a potential target for KRAS G12C combination therapy [51]. As a mediator, it can enhance signalling through the MAPK pathway. Thus, SHP2 inhibition can block RAS activation mediated by multiple RTKs. Furthermore, as a single target, SHP2 inhibition has shown favourable efficiency in preclinical models. SHP2 inhibition can reduce the level of KRAS–GTP by meditating the disruption of SOS1 and inhibiting the activation of the MAPK cascade [52]. However, single-agent SHP2 inhibition via SHP099 is incomplete. Meagan *et al*. [37] investigated the interaction between SHP2 and the RAS/MAPK pathway and revealed that the co-suppression of ARS-1620 and SHP099 can lead to complete inhibition of KRAS–GTP and RAS–GTP levels in CRC cells. This suppression of the MAPK pathway by a combination of ARS-1620 and SHP099 treatment can last much longer than SHP099 by single-agent inhibition. It was suggested that SHP2 inhibition can alleviate the induction of wild-type *RAS* and prevent the adaptive feedback resistance to KRAS G12C inhibition.

Similarly, other trials are being conducted to assess SHP2 inhibitors alone or in combination with other drugs. SHP2 inhibitors were found to effectively inhibit RTK-mediated-pathway reactivation in *KRAS-*mutated cancer [53]. Currently, Mirati *et al*. [54] are conducting a clinical trial to combination therapy with SHP2 inhibition. The selective SHP2 inhibitor RMC-4630 showed some early evidence of efficacy in patients with *KRAS G12C*-mutated NSCLC, with disease control being achieved in five of seven patients (71%). Moreover, RMC-4630 is being tested in combination with the MEK inhibitor cobimetinib, which has shown some preliminary evidence of antitumour activity in *KRAS-*mutated CRC, as indicated by tumour reduction in 37.5% of patients (three of eight). Meanwhile, Carmine *et al*. [55] demonstrated that SHP2-I/G12C-I combination can not only increase the occupancy of KRAS–GDP but also alter the immune microenvironment in *KRAS*-mutated PDAC. Similarly, Chen *et al*. [56] revealed that the novel SHP2 inhibitor TNO155 effectively blocks the feedback activation induced by KRAS G12C and substantially enhances the efficacy. The combination with TNO155 improved the efficacy of mobility shift in mutant proteins as well as decreased the rebound of p-MEK and p-ERK. *In vitro* experiments revealed that TNO155 exerts a synergistic effect with the KRAS G12C inhibitor, which can suppress the proliferation of mutant cells. In summary, combining SHP2 with the KRAS G12C inhibitor can partially reduce treatment resistance. Clinical trials of multiple specific inhibitors in combination with SHP2 are currently underway and further investigation is also warranted to determine the ideal dosage [57].

A combination of ERK and KRAS G12C inhibitors has been used to demonstrate the role of MAPK-pathway activation in resistance to KRAS G12C inhibition. Other studies have also shown that a combination of MAPK downstream pathway inhibitors can alleviate treatment resistance. MEK, a vital part of the MAPK pathway, has previously been studied. However, several clinical experiments have shown that MEK inhibition via monotherapy has limited treatment activity in patients with metastatic *KRAS*-mutated CRC [58–60]. One of the reasons for the weak effect of MEK inhibitors in *KRAS*-mutated CRC may be the activation of the collateral feedback loop [61–63]. Meanwhile, MEK inhibition plus PD-L1 inhibition has limited therapeutic efficiency in *KRAS*-mutated CRC [59]. However, a combination with other downstream inhibitions can enhance the target effect of MEK inhibition. The MEK inhibitor can synergize with the RAF inhibitor by increasing the sensitivity to RAF inhibition in *KRAS*-mutated cancer [64]. Moreover, dual inhibition of SOS1 and MEK demonstrated favourable suppression in *KRAS*-mutated mouse models [65]. Furthermore, dimerization of mutant *KRAS* and wild-type *KRAS* may affect the sensitivity to MEK inhibitors and reducing the level of wild-type *KRAS* may increase the sensitivity to MEK inhibitors in *KRAS*-mutant cells [66, 67].

KRAS has multiple downstream pathways, but inhibiting these downstream pathways is less effective than inhibiting the upfront target. This may be attributed to the complexity of the RAS pathway, in which single inhibition of a downstream target leads to the negative feedback activation of upstream targets. This activates multiple parallel signalling pathways downstream, which in turn promote tumour-cell proliferation. Hence, the combination of upstream target inhibition would be more effective than downstream inhibition alone. This strategy can block the activation of the MAPK pathway and prevent the activation of a potential parallel pathway. Further, combined inhibition of KRAS G12C and upstream targets of the MAPK pathway is crucial for minimizing treatment resistance.

### Mutation of the PI3K pathway

In addition to the combined inhibition of the MAPK pathway, the PI3K pathway plays a vital role in *KRAS*-mutated CRC. PI3K is a cytoplasmic molecule located downstream of KRAS and is a part of the PI3K/AKT/mTOR pathway. As a second effector pathway, PI3K is also activated by KRAS, in which there are three classes of PI3K activation. Class I PI3Ks, which are activated by GTP-bound RAS, phosphorylate phosphatidylinositol 4,5-bisphosphate to generate phosphatidylinositol (3,4,5)-trisphosphate. The phosphorylation of PI3K recruits AKT to the membrane and activates mTOR, which promotes cell proliferation and differentiation [68]. In contrast to the MAPK pathway, which cannot mutate simultaneously with RAS, the PI3K pathway can co-mutate with *RAS* [69]. In other words, when MAPK inhibitors or KRAS inhibitors are used alone, the PI3K pathway may be activated, resulting in therapeutic resistance. Aberrant activation of the PI3K pathway strongly reduces the efficiency of MAPK suppression in *KRAS*-mutated CRC.

Previous studies have reported that the activation of the PI3K pathway is responsible for treatment resistance to MAPK-pathway inhibitors and chemotherapy [70]. Misale *et al*. [42] revealed that the inhibition of PI3K and G12C consistently improved the suppression efficacy of various cell lines. This effect was attributed to the reactivation of ERK signalling mediated by PI3K through junctional proteins or was due to the combined shutdown of two major cellular signalling pathways [71, 72]. Some studies have demonstrated the synergistic effects of PI3K and MEK inhibitors. However, although combination therapy is more effective, it has greater toxicity [73, 74]. Further studies are needed to access the toxicity profile of multidrug combinations.

Therefore, the use of the KRAS G12C inhibitor alone is likely to lead to the activation of the PI3K pathway and the combination of PI3K and KRAS G12C inhibitors may reduce treatment resistance. However, clinical trials of PI3K and KRAS G12C in CRC have not yet been conducted. The toxicity of G12C+PI3K inhibition and its efficiency in CRC remain unclear. Further studies are needed to address these aspects using more comprehensive approaches.

### Mechanism of immune resistance

Canon *et al*. [23] revealed that AMG-510 can synergize with immunotherapy by increasing the number of infiltrating CD8^+^ T cells, thus inducing a pro-inflammatory microenvironment. Notably, AMG-510 significantly inhibited the growth of CT-26 KRAS G12C cells in immunocompetent mice; however, in mice that lacked T cells, AMG-510 only induced short-lived regression but could not treat them. This suggests that the completeness of the immune system influences KRAS G12C treatment resistance. Meanwhile, combination therapy with AMG-510 and anti-programmed cell death-ligand 1 (anti-PD-L1) showed no effect on T-cell responsiveness but had higher antitumour activity than MEK inhibitor combined with anti-PD-L1. A recent study by Ghiringhelli *et al*. [75] revealed that the combination of MEK inhibition and chemotherapy synergized with PD-L1 blockade. Furthermore, treatment with AMG-510 enhances T-cell priming and antigen recognition of tumour cells. Neither CT-26 KRAS G12C nor CT-26 parental tumours recurred after treatment with AMG-510 and anti-PD-L1, suggesting that these agents promoted the establishment of a durable immune system. Notably, Fedele *et al*. [53] also found that the KRAS G12C and SHP2 inhibitors can affect the tumour microenvironment (TME) by increasing the number of CD8^+^ T cells.

Meanwhile, a novel strategy using imaging mass cytometry revealed that the KRAS G12C inhibitor can remodel the TME and promote the infiltration and activation of presenting and effector cells [76]. These results suggest that KRAS G12C inhibitors can influence the TME of mutated cells. Future studies should explore more combinations of KRAS inhibitors with immunotherapy. Accordingly, more clinical trials combining sotorasib/adagrasib with anti-PD1/PD-L1 are currently underway (NCT03600883 and NCT03785249; clinicaltrials.gov).

### Optimizing the KRAS G12C inhibitor

**Figure 2.**
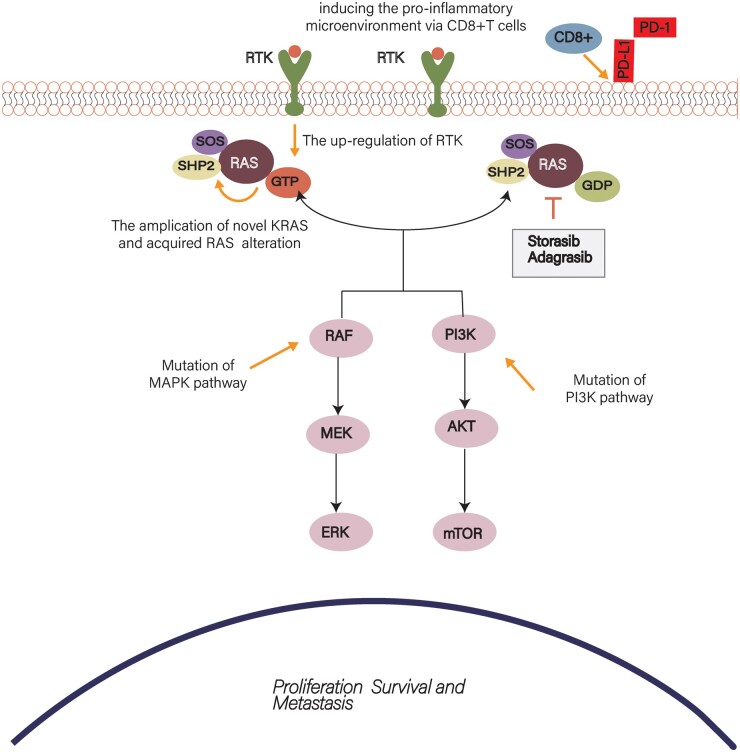
Mechanism of KRAS G12C inhibition resistance in colorectal cancer. Therapeutic resistance is achieved by inducing the activation of signalling pathways, adaptive mutation of KRAS, and alteration of the immune microenvironment. ERK, extracellular-regulated kinase; PI3K, phosphoinositide-3-kinase; MAPK, mitogen-activated protein kinase; PD-1, programmed death 1; PD-L1, programmed death-ligand 1.

**Figure 3.**
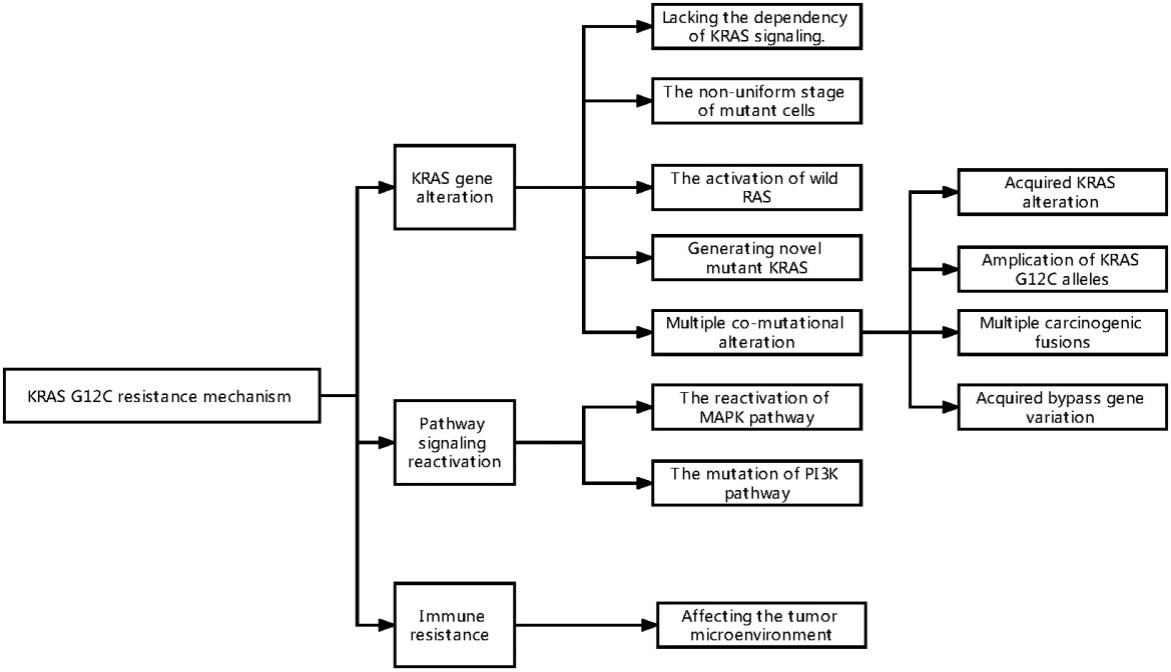
Upfront possible resistance mechanism of KRAS inhibition

Adaptive resistance feedback to KRAS G12C can result from various mechanisms, with independent or reciprocal interactions between the different mechanisms (Figure 2). Combination therapy is the best approach to overcome acquired therapeutic resistance and these commonly target upstream RTKs (especially EGFR reactivation in CRC), mediators of the MAPK cascade SHP2/SOS, adaptive alterations of *RAS* genes, and activation of multiple downstream pathways (Figure 3). In addition, the KRAS G12C target therapy can synergize with immunotherapy by remodelling the TME and enhancing the infiltration of T cells. Meanwhile, combination therapy can reduce the expression of PD-L1 and B7-H3, which enhances the antitumour immunity [77]. Compared with NSCLC, CRC is characterized by more complex tumour heterogeneity and diverse mutation mechanisms. Therefore, combination therapy with other targets is needed to achieve better tolerability in patients. The current clinical trials analysing KRAS G12C combination therapy are summarized in Table 1.

**Table 1.** Current clinical trials of KRAS G12C inhibition

Combination option	NCT#	Stage of development	Target	Estimated enrolment	Primary outcome measure
JAB-21822 + cetuximab	NCT05002270	Phase I/II recruiting	KRAS G12C (±EGFR)	100	DLTs, AEs, ORR, and DOR
JDQ443 + TNO155/tislelizumab	NCT04699188	Phase I/II recruiting	KRAS G12C (±SHP2, PD1)	375	DLTs, AEs, and ORR
MRTX849 + pembrolizumab (KRYSTAL-7)	NCT04613596	Phase II	KRAS G12C (±PDL1)	250	ORR
MRTX849 + pembrolizumab/cetuximab/afatinib (KRYSTAL-1)	NCT03785249	Phase Ib/II recruiting	KRAS G12C (±PDL1, EGFR)	740	Safety, pharmacokinetics, and clinical efficacy
MRTX849 + cetuximab (KRYSTAL-10)	NCT04793958	Phase III recruiting	KRAS G12C (+EGFR)	420	OS and PFS
LY3537982 + abemaciclib/erlotinib/sintilimab/temuterkilb/cetuximab/LY3295668	NCT04956640	Phase I recruiting	KRAS G12C (+ CDK4/6, EGFR, PD1, ERK1/2, AurA)	260	Safety and tolerability
GDC-6036 + atezolizumab/cetuximab/bevacizumab/erlotinib/GDC-1971	NCT04449874	Phase I recruiting	KRAS G12C (± PDL1, EGFR, VEGF,)	342	AEs and DLTs
LY3499446 + abemaciclib/cetuximab/erlotinib/docetaxel	NCT4165031	Terminated (due to unexpected toxicity)	KRAS G12C (± CDK4/6, EGFR, MTP)	5	DLTs, ORR, and PFS
JAB-21822 + cetuximab	NCT05194995	Phase I/II not yet recruiting	KRAS G12C (+ EGFR)	62	DLTs, ORR, and PFS
AMG510 + MVASI (vascular endothelial growth factor)	NCT05180422	Phase I/II not yet recruiting	KRAS G12C (+ VEGF)	43	Dose exploration
AMG510 + anti-PD-1/L1 (CodeBreak100)	NCT03600883	Phase I/II recruiting	KRAS G12C (± PD1/PD-L1)	733	DLTs, ORR, SD, TTR, and clinical change
AMG510 + VS-6766 (RAMP203)	NCT05074810	Phase I/II not yet recruiting	KRAS G12C (+ MEK/RAF)	53	DLTs and ORR
AMG510 + AMG-404/trametinib/RMC-4630/afatinib/pembro/panitumumab/carbo/pem/docetaxel/everolimus/palbociclib/bevacizumab/adagrasib/TNO155 (CodeBreak101)	NCT04185883	Phase I/II recruiting	KRAS G12C (+ PD-1/MEK/SHP2/pan-ErbB)	1,280	DLTs, TEAES, ORR, and clinical change
AMG510 + cisplatin/carboplatin/pemetrexed	NCT05118854	Phase II not yet recruiting	KRAS G12C + chemotherapy	27	PRR
MRTX849 + BI1701963 (KRYSTAL14)	NCT04975256	Phase I recruiting	KRAS G12C (+ SOS1)	100	AEs, blood-plasma concentration, and maximum tolerated dose
MRTX849 + TNO155	NCT04330664	Phase II active	KRAS G12C (+ SHP2)	86	AEs and blood-plasma concentration
MRTX849 + palbociclib (KRYSTAL-16)	NCT05178888	Phase I recruiting	KRAS G12C (+ CDK4/6)	50	AEs, blood-plasma concentration, maximum tolerated dose, and ORR
BI1823911 + BI1701963	NCT04973163	Phase I recruiting	KRAS G12C (+ KRAS G12C)	60	DLTs and OR
AMG510 + nal-IRI/5FU/LV/GEM/nab paclitaxel	NCT05251038	Phase I/II not yet recruiting	KRAS G12C + chemotherapy	59	ORR
AMG510 + RMC-4630	NCT050554725	Phase II recruiting	KRAS G12C (+ SHP2)	46	ORR
AMG510 + panitumumab (CodeBreak300)	NCT05198934	Phase III not yet recruiting	KRAS G12C (+ EGFR)	153	PFS

KRAS, Kirsten rat sarcoma viral oncogene homologue; DLT, dose-limiting toxicity; AEs, adverse events; ORR, overall response rate; DOR, duration of response; PFS, progression-free survival; SD, stable disease; TTR, time to response; TEAES, treatment-emergent adverse events.

## Conclusions

Current knowledge regarding the KRAS G12C inhibitor debunks the notion that the *KRAS* oncogene is ‘undruggable’. Numerous targeted agents that are successfully marketed have demonstrated both efficacy and tolerability [28, 29]. However, in *BRAF*- and *EGFR*-mutated CRC, monotherapy will ultimately lead to the emergence of drug resistance due to adaptive resistance or mutation of the gene itself. Furthermore, the resistance mechanism is tissue-specific and, among the above reasons, the activation of the upstream EGFR pathway is the primary factor in CRC. It should be emphasized that KRAS and its upstream and downstream pathways such as MAPK and PI3K are not singularly regulated, and multiple targets and downstream pathways can be mediated by negative feedback activation. This may lead to the reactivation of downstream pathways, resulting in drug resistance. Meanwhile, the variation in KRAS G12C inhibitors in diverse populations and tumour types also indicate the existence of intrinsic resistance mechanisms [78].

The commercial availability of KRAS G12C inhibitor should also be considered. *G12C* mutation accounts for only 3% of all mutations in patients with CRC. Some patients can benefit from this target therapy; however, acquired resistance and real-world therapeutic effects are still challenges for G12C target therapy as other patients with *KRAS* mutation still need more effective target methods. Therefore, searching more specific biomarkers and broader target inhibitors and comprehensively utilizing multi-pathway inhibition (e.g. co-targeting molecules in various pathways such as SOS/SHP2) are important strategies to alleviate KRAS treatment resistance, which warrants further investigation.

## Authors’ Contributions

A.W.W. conceived and designed the project. M.H.Z. and A.W.W. wrote the manuscript, as well as prepared the tables and pictures. All authors read and approved the final manuscript.

## References

[1] Hobbs GA , Der CJ, Rossman KL. RAS isoforms and mutations in cancer at a glance. J Cell Sci 2016;129:1287–92. 26985062 10.1242/jcs.182873 PMC4869631

[2] Vögler O , Barceló JM, Ribas C et al Membrane interactions of G proteins and other related proteins. Biochim Biophys Acta 2008;1778:1640–52. 18402765 10.1016/j.bbamem.2008.03.008

[3] Lowy DR , Willumsen BM. Function and regulation of RAS. Annu Rev Biochem 1993;62:851–91. 8352603 10.1146/annurev.bi.62.070193.004223

[4] Adjei AA. Blocking oncogenic RAS signaling for cancer therapy. J Natl Cancer Inst 2001;93:1062–74. 11459867 10.1093/jnci/93.14.1062

[5] Drosten M , Dhawahir A, Sum EY et al Genetic analysis of RAS signalling pathways in cell proliferation, migration and survival. EMBO J 2010;29:1091–104. 20150892 10.1038/emboj.2010.7 PMC2845279

[6] Suman S , Kurisetty V, Das TP et al Activation of AKT signaling promotes epithelial-mesenchymal transition and tumor growth in colorectal cancer cells. Mol Carcinog 2014;53(Suppl 1):E151–60. 24000138 10.1002/mc.22076

[7] Rychahou PG , Kang J, Gulhati P et al Akt2 overexpression plays a critical role in the establishment of colorectal cancer metastasis. Proc Natl Acad Sci USA 2008;105:20315–20. 19075230 10.1073/pnas.0810715105 PMC2629319

[8] Gao T , Wang M, Xu L et al DCLK1 is up-regulated and associated with metastasis and prognosis in colorectal cancer. J Cancer Res Clin Oncol 2016;142:2131–40. 27520310 10.1007/s00432-016-2218-0 PMC11819345

[9] Sun Y , Ji B, Feng Y et al TRIM59 facilitates the proliferation of colorectal cancer and promotes metastasis via the PI3K/AKT pathway. Oncol Rep 2017;38:43–52. 28534983 10.3892/or.2017.5654 PMC5492839

[10] Gehlenborg N , Research T, Atlas T et al Cancer Genome Atlas Research Network. Comprehensive molecular characterization of human colon and rectal cancer. Nature 2012;18:330–7. 10.1038/nature11252 PMC3401966 22810696

[11] Bailey P , Chang DK, Nones K et al; Australian Pancreatic Cancer Genome Initiative. Genomic analyses identify molecular subtypes of pancreatic cancer. Nature 2016;531:47–52. 26909576 10.1038/nature16965

[12] Campbell JD , Alexandrov A, Kim J et al; Cancer Genome Atlas Research Network. Distinct patterns of somatic genome alterations in lung adenocarcinomas and squamous cell carcinomas. Nat Genet 2016;48:607–16. 27158780 10.1038/ng.3564 PMC4884143

[13] Vasan N , Boyer JL, Herbst RS. A RAS renaissance: emerging targeted therapies for KRAS-mutated non-small cell lung cancer. Clin Cancer Res 2014;20:3921–30. 24893629 10.1158/1078-0432.CCR-13-1762 PMC5369356

[14] Hayama T , Hashiguchi Y, Okamoto K et al G12V and G12C mutations in the gene KRAS are associated with a poorer prognosis in primary colorectal cancer. Int J Colorectal Dis 2019;34:1491–6. 31309326 10.1007/s00384-019-03344-9

[15] Cremolini C , Loupakis F, Antoniotti C et al FOLFOXIRI plus bevacizumab versus FOLFIRI plus bevacizumab as first-line treatment of patients with metastatic colorectal cancer: updated overall survival and molecular subgroup analyses of the open-label, phase 3 TRIBE study. Lancet Oncol 2015;16:1306–15. 26338525 10.1016/S1470-2045(15)00122-9

[16] Xue JY , Zhao Y, Aronowitz J et al Rapid non-uniform adaptation to conformation-specific KRAS(G12C) inhibition. Nature 2020;577:421–5. 31915379 10.1038/s41586-019-1884-x PMC7308074

[17] Scheffzek K , Ahmadian MR, Kabsch W et al The RAS-RASGAP complex: structural basis for GTPase activation and its loss in oncogenic RAS mutants. Science 1997;277:333–8. 9219684 10.1126/science.277.5324.333

[18] Bourne HR , Sanders DA, McCormick F. The GTPase superfamily: conserved structure and molecular mechanism. Nature 1991;349:117–27. 1898771 10.1038/349117a0

[19] Santos E , Nebreda AR. Structural and functional properties of RAS proteins. FASEB J 1989;3:2151–63. 2666231 10.1096/fasebj.3.10.2666231

[20] Riely GJ , Marks J, Pao W. KRAS mutations in non-small cell lung cancer. Proc Am Thorac Soc 2009;6:201–5. 19349489 10.1513/pats.200809-107LC

[21] Cox AD , Fesik SW, Kimmelman AC et al Drugging the undruggable RAS: mission possible? Nat Rev Drug Discov 2014;13:828–51. 25323927 10.1038/nrd4389 PMC4355017

[22] Ryan MB , Corcoran RB. Therapeutic strategies to target RAS-mutant cancers. Nat Rev Clin Oncol 2018;15:709–20. 30275515 10.1038/s41571-018-0105-0

[23] Canon J , Rex K, Saiki AY et al The clinical KRAS(G12C) inhibitor AMG 510 drives anti-tumour immunity. Nature 2019;575:217–23. 31666701 10.1038/s41586-019-1694-1

[24] Ostrem JM , Shokat KM. Direct small-molecule inhibitors of KRAS: from structural insights to mechanism-based design. Nat Rev Drug Discov 2016;15:771–85. 27469033 10.1038/nrd.2016.139

[25] Patricelli MP , Janes MR, Li LS et al Selective inhibition of oncogenic KRAS output with small molecules targeting the inactive state. Cancer Discov 2016;6:316–29. 26739882 10.1158/2159-8290.CD-15-1105

[26] Janes MR , Zhang J, Li LS et al Targeting KRAS mutant cancers with a covalent G12C-specific inhibitor. Cell 2018;172:578–89.e17. 29373830 10.1016/j.cell.2018.01.006

[27] Hallin J , Engstrom LD, Hargis L et al The KRAS(G12C) inhibitor MRTX849 provides insight toward therapeutic susceptibility of KRAS-mutant cancers in mouse models and patients. Cancer Discov 2020;10:54–71. 31658955 10.1158/2159-8290.CD-19-1167 PMC6954325

[28] Hong DS , Fakih MG, Strickler JH et al KRAS(G12C) inhibition with sotorasib in advanced solid tumors. N Engl J Med 2020;383:1207–17. 32955176 10.1056/NEJMoa1917239 PMC7571518

[29] Skoulidis F , Li BT, Dy GK et al Sotorasib for lung cancers with KRAS p.G12C mutation. N Engl J Med 2021;384:2371–81. 34096690 10.1056/NEJMoa2103695 PMC9116274

[30] Lindsay CR , Blackhall FH. Direct RAS G12C inhibitors: crossing the rubicon. Br J Cancer 2019;121:197–8. 31239544 10.1038/s41416-019-0499-1 PMC6738074

[31] Johnson ML , Sai-Hong IO, Barve M, Rybkin II, KRYSTAL-1: activity and safety of adagrasib (MRTX849) in patients with colorectal cancer and other solid tumors harboring a KRAS G12C mutation. In: EORTC-NCI-AACR Symposium, Vol. 138 2020; Abstract LBA4, European Journal of Cancer. pp. S2.

[32] Corcoran RB , André T, Atreya CE et al Combined BRAF, EGFR, and MEK inhibition in patients with BRAF(V600E)-mutant colorectal cancer. Cancer Discov 2018;8:428–43. 29431699 10.1158/2159-8290.CD-17-1226 PMC5882509

[33] Planchard D , Besse B, Groen HJM et al Dabrafenib plus trametinib in patients with previously treated BRAF(V600E)-mutant metastatic non-small cell lung cancer: an open-label, multicentre phase 2 trial. Lancet Oncol 2016;17:984–93. 27283860 10.1016/S1470-2045(16)30146-2 PMC4993103

[34] Jiao D , Yang S. Overcoming resistance to drugs targeting KRAS(G12C) mutation. Innovation (N Y) 2020;1:100035. 10.1016/j.xinn.2020.100035 PMC7491749 32939510

[35] Jänne PA , Rybkin II, Spira AI et al KRYSTAL-1: activity and safety of adagrasib (MRTX849) in advanced/metastatic non-small-cell lung cancer (NSCLC) harboring KRAS G12C mutation. Eur J Cancer 2020;138:S1–S2.

[36] Muzumdar MD , Chen PY, Dorans KJ et al Survival of pancreatic cancer cells lacking KRAS function. Nat Commun 2017;8:1090. 29061961 10.1038/s41467-017-00942-5 PMC5653666

[37] Ryan MB , Fece de la Cruz F, Phat S et al Vertical pathway inhibition overcomes adaptive feedback resistance to KRAS(G12C) inhibition. Clin Cancer Res 2020;26:1633–43. 31776128 10.1158/1078-0432.CCR-19-3523 PMC7124991

[38] Zhao Y , Murciano-Goroff YR, Xue JY et al Diverse alterations associated with resistance to KRAS(G12C) inhibition. Nature 2021;599:679–83. 34759319 10.1038/s41586-021-04065-2 PMC8887821

[39] Awad MM , Liu S, Rybkin II et al Acquired resistance to KRAS(G12C) inhibition in cancer. N Engl J Med 2021;384:2382–93. 34161704 10.1056/NEJMoa2105281 PMC8864540

[40] Koga T , Suda K, Fujino T et al KRAS secondary mutations that confer acquired resistance to KRAS G12C inhibitors, sotorasib and adagrasib, and overcoming strategies: insights from in vitro experiments. J Thorac Oncol 2021;16:1321–32. 33971321 10.1016/j.jtho.2021.04.015

[41] Tanaka N , Lin JJ, Li C et al Clinical acquired resistance to KRAS(G12C) inhibition through a novel KRAS switch-II pocket mutation and polyclonal alterations converging on RAS-MAPK reactivation. Cancer Discov 2021;11:1913–22. 33824136 10.1158/2159-8290.CD-21-0365 PMC8338755

[42] Misale S , Fatherree JP, Cortez E et al KRAS G12C NSCLC models are sensitive to direct targeting of KRAS in combination with PI3K inhibition. Clin Cancer Res 2019;25:796–807. 30327306 10.1158/1078-0432.CCR-18-0368

[43] Prahallad A , Sun C, Huang S et al Unresponsiveness of colon cancer to BRAF(V600E) inhibition through feedback activation of EGFR. Nature 2012;483:100–3. 22281684 10.1038/nature10868

[44] Lito P , Pratilas CA, Joseph EW et al Relief of profound feedback inhibition of mitogenic signaling by RAF inhibitors attenuates their activity in BRAFV600E melanomas. Cancer Cell 2012;22:668–82. 23153539 10.1016/j.ccr.2012.10.009 PMC3713778

[45] Amodio V , Yaeger R, Arcella P et al EGFR blockade reverts resistance to KRAS(G12C) inhibition in colorectal cancer. Cancer Discov 2020;10:1129–39. 32430388 10.1158/2159-8290.CD-20-0187 PMC7416460

[46] Misale S , Yaeger R, Hobor S et al Emergence of KRAS mutations and acquired resistance to anti-EGFR therapy in colorectal cancer. Nature 2012;486:532–6. 22722830 10.1038/nature11156 PMC3927413

[47] Rosell R , Carcereny E, Gervais R et al; Spanish Lung Cancer Group in collaboration with Groupe Français de Pneumo-Cancérologie and Associazione Italiana Oncologia Toracica. Erlotinib versus standard chemotherapy as first-line treatment for European patients with advanced EGFR mutation-positive non-small-cell lung cancer (EURTAC): a multicentre, open-label, randomised phase 3 trial. Lancet Oncol 2012;13:239–46. 22285168 10.1016/S1470-2045(11)70393-X

[48] Soria JC , Ohe Y, Vansteenkiste J et al; FLAURA Investigators. Osimertinib in untreated EGFR-mutated advanced non-small-cell lung cancer. N Engl J Med 2018;378:113–25. 29151359 10.1056/NEJMoa1713137

[49] Qin S , Li J, Wang L et al Efficacy and tolerability of first-line cetuximab plus leucovorin, fluorouracil, and oxaliplatin (FOLFOX-4) versus FOLFOX-4 in patients with RAS wild-type metastatic colorectal cancer: the open-label, randomized, phase III TAILOR trial. J Clin Oncol 2018;36:3031–9. 30199311 10.1200/JCO.2018.78.3183 PMC6324088

[50] Kopetz S , Grothey A, Yaeger R et al Encorafenib, binimetinib, and cetuximab in BRAF V600E-mutated colorectal cancer. N Engl J Med 2019;381:1632–43. 31566309 10.1056/NEJMoa1908075

[51] Dance M , Montagner A, Salles JP et al The molecular functions of Shp2 in the RAS/Mitogen-activated protein kinase (ERK1/2) pathway. Cell Signal 2008;20:453–9. 17993263 10.1016/j.cellsig.2007.10.002

[52] Nichols RJ , Haderk F, Stahlhut C et al RAS nucleotide cycling underlies the SHP2 phosphatase dependence of mutant BRAF-, NF1- and RAS-driven cancers. Nat Cell Biol 2018;20:1064–73. 30104724 10.1038/s41556-018-0169-1 PMC6115280

[53] Fedele C , Ran H, Diskin B et al SHP2 inhibition prevents adaptive resistance to MEK inhibitors in multiple cancer models. Cancer Discov 2018;8:1237–49. 30045908 10.1158/2159-8290.CD-18-0444 PMC6170706

[54] Ou SI , Koczywas M, Ulahannan S et al A12 the SHP2 inhibitor RMC-4630 in patients with KRAS-mutant non-small cell lung cancer: preliminary evaluation of a first-in-man phase 1 clinical trial. J Thorac Oncol 2020;15:S15–6.

[55] Fedele C , Li S, Teng KW et al SHP2 inhibition diminishes KRASG12C cycling and promotes tumor microenvironment remodeling. J Exp Med 2021;218:e20201414. 10.1084/jem.20201414 PMC7549316 33045063

[56] Liu C , Lu H, Wang H et al Combinations with allosteric SHP2 inhibitor TNO155 to block receptor tyrosine kinase signaling. Clin Cancer Res 2021;27:342–54. 33046519 10.1158/1078-0432.CCR-20-2718

[57] Hata AN , Shaw AT. Resistance looms for KRAS(G12C) inhibitors. Nat Med 2020;26:169–70. 32020086 10.1038/s41591-020-0765-z

[58] Bennouna J , Lang I, Valladares-Ayerbes M et al A Phase II, open-label, randomised study to assess the efficacy and safety of the MEK1/2 inhibitor AZD6244 (ARRY-142886) versus capecitabine monotherapy in patients with colorectal cancer who have failed one or two prior chemotherapeutic regimens. Invest New Drugs 2011;29:1021–8. 20127139 10.1007/s10637-010-9392-8

[59] Hellmann MD , Kim TW, Lee CB et al Phase Ib study of atezolizumab combined with cobimetinib in patients with solid tumors. Ann Oncol 2019;30:1134–42. 30918950 10.1093/annonc/mdz113 PMC6931236

[60] Zimmer L , Barlesi F, Martinez-Garcia M et al Phase I expansion and pharmacodynamic study of the oral MEK inhibitor RO4987655 (CH4987655) in selected patients with advanced cancer with RAS-RAF mutations. Clin Cancer Res 2014;20:4251–61. 24947927 10.1158/1078-0432.CCR-14-0341

[61] Friday BB , Yu C, Dy GK et al BRAF V600E disrupts AZD6244-induced abrogation of negative feedback pathways between extracellular signal-regulated kinase and RAF proteins. Cancer Res 2008;68:6145–53. 18676837 10.1158/0008-5472.CAN-08-1430

[62] Hatzivassiliou G , Haling JR, Chen H et al Mechanism of MEK inhibition determines efficacy in mutant KRAS- versus BRAF-driven cancers. Nature 2013;501:232–6. 23934108 10.1038/nature12441

[63] Lito P , Saborowski A, Yue J et al Disruption of CRAF-mediated MEK activation is required for effective MEK inhibition in KRAS mutant tumors. Cancer Cell 2014;25:697–710. 24746704 10.1016/j.ccr.2014.03.011 PMC4049532

[64] Yen I , Shanahan F, Merchant M et al Pharmacological induction of RAS-GTP confers RAF inhibitor sensitivity in KRAS mutant tumors. Cancer Cell 2018;34:611–25.e7. 30300582 10.1016/j.ccell.2018.09.002

[65] Hofmann MH , Gmachl M, Ramharter J et al BI-3406, a potent and selective SOS1-KRAS interaction inhibitor, is effective in KRAS-driven cancers through combined MEK inhibition. Cancer Discov 2021;11:142–57. 32816843 10.1158/2159-8290.CD-20-0142 PMC7892644

[66] Burgess MR , Hwang E, Mroue R et al KRAS allelic imbalance enhances fitness and modulates MAP kinase dependence in cancer. Cell 2017;168:817–29.e15. 28215705 10.1016/j.cell.2017.01.020 PMC5541948

[67] Ambrogio C , Köhler J, Zhou ZW et al KRAS dimerization impacts MEK inhibitor sensitivity and oncogenic activity of mutant KRAS. Cell 2018;172:857–68.e15. 29336889 10.1016/j.cell.2017.12.020

[68] Fruman DA , Chiu H, Hopkins BD et al The PI3K pathway in human disease. Cell 2017;170:605–35. 28802037 10.1016/j.cell.2017.07.029 PMC5726441

[69] Cancer Genome Atlas Research Network. Comprehensive molecular profiling of lung adenocarcinoma. Nature 2014;511:543–50. 25079552 10.1038/nature13385 PMC4231481

[70] Wang Q , Shi YL, Zhou K et al PIK3CA mutations confer resistance to first-line chemotherapy in colorectal cancer. Cell Death Dis 2018;9:739. 29970892 10.1038/s41419-018-0776-6 PMC6030128

[71] Engelman JA , Chen L, Tan X et al Effective use of PI3K and MEK inhibitors to treat mutant Kras G12D and PIK3CA H1047R murine lung cancers. Nat Med 2008;14:1351–6. 19029981 10.1038/nm.1890 PMC2683415

[72] Alagesan B , Contino G, Guimaraes AR et al Combined MEK and PI3K inhibition in a mouse model of pancreatic cancer. Clin Cancer Res 2015;21:396–404. 25348516 10.1158/1078-0432.CCR-14-1591 PMC4447091

[73] Shimizu T , Tolcher AW, Papadopoulos KP et al The clinical effect of the dual-targeting strategy involving PI3K/AKT/mTOR and RAS/MEK/ERK pathways in patients with advanced cancer. Clin Cancer Res 2012;18:2316–25. 22261800 10.1158/1078-0432.CCR-11-2381

[74] Juric D , Soria JC, Sharma S et al A phase 1b dose-escalation study of BYL719 plus binimetinib (MEK162) in patients with selected advanced solid tumors. J Clin Oncol 2014;32:9051.

[75] Limagne E , Nuttin L, Thibaudin M et al MEK inhibition overcomes chemoimmunotherapy resistance by inducing CXCL10 in cancer cells. Cancer Cell 2022;40:136–52.e12. 35051357 10.1016/j.ccell.2021.12.009

[76] van Maldegem F , Valand K, Cole M et al Characterisation of tumour microenvironment remodelling following oncogene inhibition in preclinical studies with imaging mass cytometry. Nat Commun 2021;12:5906. 34625563 10.1038/s41467-021-26214-x PMC8501076

[77] Ward AB , Keeton AB, Chen X et al Enhancing anticancer activity of checkpoint immunotherapy by targeting RAS. MedComm (2020) 2020;1:121–8. 33073260 10.1002/mco2.10 PMC7567124

[78] Huang L , Guo Z, Wang F et al KRAS mutation: from undruggable to druggable in cancer. Signal Transduct Target Ther 2021;6:386. 34776511 10.1038/s41392-021-00780-4 PMC8591115

